# Evaluation of hormonal and circulating inflammatory biomarker profiles in the year following bariatric surgery

**DOI:** 10.3389/fendo.2023.1171675

**Published:** 2023-07-25

**Authors:** Eun Ran Kim, Ji Ho Yun, Hyo-Jin Kim, Hyeon Young Park, Yoonseok Heo, Young Suk Park, Do Joong Park, Soo Kyung Koo

**Affiliations:** ^1^ Division of Endocrine and Kidney Disease Research, Department of Chronic Disease Convergence, Korea National Institute of Health, Cheongju-si, Chungbuk, Republic of Korea; ^2^ Department of Surgery, School of Medicine, Inha University, Incheon, Republic of Korea; ^3^ Department of Surgery, Seoul National University Bundang Hospital, Seongnam, Republic of Korea; ^4^ Department of Surgery, Seoul National University Hospital, Seoul, Republic of Korea

**Keywords:** obesity, bariatric surgery, diabetes, hormone, inflammation

## Abstract

**Background:**

Bariatric surgery (BS) has a superior effect on reducing body weight and fat in patients with morbid obesity. As a result, BS mitigates obesity-related complications such as type 2 diabetes (T2D). However, few studies have shown the mechanism underlying diabetes remission after surgery. This study aimed to investigate the differences in serum hormone and inflammatory cytokine levels related to diabetes before surgery and during 12 months of follow-up in Korean patients with obesity.

**Methods:**

The study participants were patients with morbid obesity (n=63) who underwent sleeve gastrectomy (SG) or Roux-en-Y gastric bypass (RYGB) between 2016 – 2017 at seven tertiary hospitals in Korea. The patients were followed for 1 year after surgery.

**Results:**

Sixty-three patients had significant weight loss after surgery and showed improvements in clinical parameters and hormonal and inflammatory profiles. Among them, 23 patients who were diabetic preoperatively showed different remission after surgery. The levels of inflammation-related clinical parameters changed significantly in the remission group, and serum inflammatory cytokine and hormones significantly decreased at certain points and showed an overall decreasing trend.

**Conclusions:**

Our study found postoperative changes of factors in blood samples, and the changes in hormones secreted from the three major metabolic tissue (pancreas, adipose, and gut) along with the differences in multi-origin inflammatory cytokines between remission and non-remission groups provide a path for understanding how the effect of BS in improving glucose metabolism is mediated.

## 1 Introduction

Compared to pharmacotherapy in the treatment of obesity (1), bariatric surgery (BS) has a superior effect on reducing body weight and fat in patients with severe obesity. Furthermore, it mitigates type 2 diabetes, hypertension, and dyslipidemia. Roux-en-Y gastric bypass (RYGB) and sleeve gastrectomy (SG), which change the anatomical structure of the gastrointestinal (GI) tract and hormone levels, are the most performed types of BS (2–4). Postprandial hormone responses, such as that of glucagon-like peptide 1 (GLP-1), stimulate insulin secretion and lower glucose levels (5). Obese subjects have high serum levels of GLP-1 (6), indicating that the malfunction of GLP-1 signaling can cause insulin resistance. Patients who underwent BS were also reported to have increased postprandial GLP-1 levels (7). Leptin is a hormone produced by adipocytes and leptin levels have a strong correlation with obesity severity. In these cases, hyperleptinemia is usually observed, but it decreases with decreasing body weight. Thus, leptin levels can also be affected by BS. However, GI hormones are not the only ones affected by BS since it also modulates inflammatory cytokines.

Chronic inflammation in obese patients plays an important role in the development of metabolic diseases and increases the risk of complications such as hypertension, insulin resistance, atherosclerosis, chronic kidney disease, and aging-related diseases such as sarcopenia (8–10). Cytokines and hormones originating from different sites contribute to the development of tissue inflammation (11, 12). For example, interleukin-1β (IL-1β) and monocyte chemoattractant protein-1 (MCP-1) increase, and MCP-1 promotes macrophage infiltration in metabolic tissues (13).

Although BS has shown promising effects in patients with severe obesity and type 2 diabetes mellitus (T2D), not all patients who undergo BS show improvements in glucose regulation. A proportion of patients (approximately 10-67%) with BS still experience dysregulation of glucose metabolism (14–16), even though their body weights were significantly lower. The reasons why some patients experience diabetes remission while others do not after BS remain unclear. Few studies have been conducted on the effects of BS in Korean patients.

Recently, owing to its acceptance by the Korean population and coverage by medical insurance, more patients have been undergoing BS. However, few studies have been conducted on Korean patients. There are several important differences in terms of ethnic background: i) the rates of body fat content over body mass are higher in Asian populations than in Western populations (17), ii) an improvement of diabetes condition has advanced to substantial reduction of body weight (18, 19), and iii) beta cell dysfunction is more severe in Asian populations including Korean than in Western populations with the same BMI (20, 21). Therefore, it is important to establish data on Korean patients. Many researchers and clinicians have attempted to divide patients into subtypes including ethnic backgrounds for a more effective and personalized approach to therapy.

In this study, we assessed hormone and inflammatory cytokine levels up to 12 months after RYGB or SG in Korean patients. We further divided the patients into diabetes remission and non-remission groups and monitored changes in hormone and inflammatory cytokine levels.

## 2 Materials and methods

### 2.1 Study participants

The study participants and study design were previously described in the Korean Obesity Surgical Treatment Study (KOBESS) (1) and registered at ClinicalTrials.gov (NCT03100292). Briefly, this was a prospective, multicenter, single-arm, observational cohort study of obese Korean patients with obesity. Sixty-three participants were recruited between 2016 – 2017 at seven tertiary hospitals in Korea. Sleeve gastrectomy (SG) or Roux-en-Y gastric bypass (RYGB) surgical methods were conducted on the participants, and they were followed for 1 year after surgery. All consecutive patients aged 20 – 65 years with a body mass index (BMI) of ≥ 35 kg/m^2^ or a BMI of ≥ 30 kg/m^2^ with comorbid conditions, including hypertension, glucose intolerance, dyslipidemia, or obstructive sleep apnea (OSA) were included. The study was approved by the Korea Disease Control and Prevention Agency (KDCA) and Inha University Hospital Institutional Review Board (2021-10-08-P-A and 2019-10-025-002, respectively). All participants provided written informed consent.

### 2.2 Type 2 diabetes remission subgroup

The complete remission of T2D was defined as a glycated hemoglobin (HbA1c) level <6% and a fasting blood sugar (FBS) level <100 mg/dL for 1 year; partial remission was defined as HbA1c<6.5% and FBS 100–125 mg/dL, without anti-diabetes medication use for 1 year after the surgery. Diabetes medication was stopped in all patients postoperatively. However, if the HbA1C exceeded 7% during the follow up period, anti-diabetes medication was restarted. Non-remission group included partial remission group as well as the patients who did not achieve any remission criteria (22). The study for remission of T2D subject, participants (remission n = 11, non-remission n =12) were collected from KOBESS study as previously described (1). Clinical and serum parameters were analyzed up to 12 months after surgery.

### 2.3 Serum profiling

Firstly, we selected specific time points for measuring hormone levels: before the surgery and at 1, 6, and 12 months after the procedure, in order to examine the immediate changes following surgery (at 1 month) and track the progress every 6 months thereafter. Subsequently, considering the observed changes in hormone levels, we conducted additional investigations into the levels of inflammatory factors. This analysis aimed to determine if cytokine changes occurred prior to hormonal changes within the 6-month period following surgery. We examined the levels of inflammatory factors at 3 months post-surgery, opting for this time point instead of the standard 6 months. Serum samples were analyzed with a Luminex instrument (Luminex, Austin, TX) using MILLIPLEX^®^ multiplex panels (Millipore Corporation, Billerica, MA, USA) and performed by LAS Inc, South Korea (https://www.las.kr) according to the manufacturer’s instructions. All panels available for the analysis of human hormones or inflammatory factors were provided by Millipore, including HMHEMAG-34K (GLP-1, GIP, insulin, leptin, glucagon, and amylin), HADK1MAG-61K (adiponectin and resistin), and HCYTOMAG-60K (IL-1β, IL-Ra, IL-4, IL-6, IL-17A, MCP1, tumor necrosis factor (TNF)α, TNFβ, transforming growth factor (TGF)α, TNFβ, Granulocyte colony-stimulating factor (G-CSF) and Platelet-Derived Growth Factor (PDGF) AA). The concentration of hormones or inflammatory factors was calculated using a 5-parameter logistic or cube spline standard curve and expressed as pg/mL or ng/mL.

### 2.4 Statistics analysis

Statistical analyses were conducted using SAS statistical software package (version 9.4; SAS Institute, Inc., Cary, NC, USA) or Prism 8.3.0 (GraphPad Software, San Diego, CA, USA). The values are expressed as means ± standard deviation (SD) for continuous variables and numbers (%) for categorical variables. The significant differences between baseline and postoperative time points were determined using one-way analysis of variance (ANOVA, mixed-effect analysis) followed by a paired t-test with Dunnett's multiple comparisons tests. For all analyses, p-values of < 0.05 were considered statistically significant.

## 3 Results

### 3.1 Patient demographics and clinical characteristics at baseline

Sixty-three patients underwent BS during the study period. Twenty-one patients underwent RYGB, and 42 underwent SG. The clinical baselines and characteristics of the study participants are summarized in **Table 1**. The mean age for the entire cohort was 37.5 ± 11.8 years old. The mean BMI of the patients was 38.9 ± 6.7 kg/m^2^, indicating class II obesity, which was defined as 35 kg/m^2^ ≤ BMI ≤ 39.99 kg/m^2^ by the International Classification of Disease (ICD). Twenty-three patients (36.5%) had diabetes, and 49 (77.8%) and 37 (58.7%) had hypertension and dyslipidemia, respectively.

**Table 1:** Baseline characteristics of patients with bariatric surgery.

Characteristics	*n*	Total (*N*=63)	
Sex, n(%)	63		
Men		19	(30.2)
Women		44	(69.8)
Age (years)	63	37.5	± 11.8
Weight (kg)	63	108.8	± 25.7
BMI (kg/m^2^)	63	38.9	± 6.7
Waist circumference (cm)	59	119.3	± 16.3
WHR (Waist-Hip Ratio)	59	0.97	± 0.06
SBP (mmHg)	62	142.0	± 17.9
DBP (mmHg)	62	87.7	± 15.0
Fasting glucose (mg/dl)	62	123.9	± 45.3
Total cholesterol (mg/dl)	63	192.8	± 37.8
Triglyceride (mg/dl)	63	167.5	± 135.3
HDL-cholesterol (mg/dl)	63	48.8	± 12.8
LDL-cholesterol (mg/dl)	62	118.8	± 28.8
Clinical history, n(%)			
Type 2 diabetes	63	23	(36.5)
Hypertension	63	49	(77.8)
Dyslipidemia	63	37	(58.7)
Bariatric surgery, n(%)	63		
Roux-e-Y Gastric Bypass		21	(33.3)
Sleeve Gastrectomy		42	(66.7)

Values are presented as the mean ± SD for continuous variables and n (%) for categorical variables.

BMI, body mass index; SD, standard deviation.

### 3.2 Post-surgery changes during 12 months of follow-up

Bariatric surgery induced a mean weight loss of 10.5 ± 4.5 kg over 1 month and 31.3 ± 4.2 kg over 12 months. Likewise, a significant reduction in BMI was observed starting 1 month after surgery (**Table 2**).

**Table 2:** Comparison of clinical characteristic between before and after bariatric surgery.

	Baseline			Postoperative															p-value^1)^	p-value^2)^	p-value^3)^	p-value^4)^	p-value^5)^
				1 week			1 month			3 months			6 months			12 months							
	*N*	Mean	±SD	*N*	Mean	±SD	*N*	Mean	±SD	*N*	Mean	±SD	*N*	Mean	±SD	*N*	Mean	±SD					
**Weight (kg)**	63	108.8	± 25.7	61	109.0	± 25.4	60	98.3	± 24.1	59	88.5	± 22.9	56	81.8	± 21.5	58	77.5	± 19.9	0.174	<.0001^****^	<.0001^****^	<.0001^****^	<.0001^****^
**BMI (kg/m^2^)**	63	38.9	± 6.7	61	38.9	± 6.5	60	35.1	± 6.3	59	31.5	± 5.8	56	29.1	± 5.6	58	27.6	± 5.3	0.142	<.0001^****^	<.0001^****^	<.0001^****^	<.0001^****^
**Waist circumference (cm)**	59	119.3	± 16.3	*NA*			*NA*			53	103.3	± 16.7	55	96.4	± 14.4	50	92.4	± 16.7	*NA*	*NA*	<.0001^****^	<.0001^****^	<.0001^****^
**Hip**	59	122.6	± 13.9	*NA*			*NA*			51	110.6	± 12.9	55	105.9	± 11.9	50	102.8	± 14.6	*NA*	*NA*	<.0001^****^	<.0001^****^	<.0001^****^
**WHR(Waist-Hip Ratio)**	59	0.97	± 0.06	*NA*			*NA*			51	0.93	± 0.07	55	0.91	± 0.07	50	0.90	± 0.07	*NA*	*NA*	<.0001^****^	<.0001^****^	<.0001^****^
**SBP (mmHg)**	62	142.0	± 17.9	61	128.3	± 13.8	58	128.8	± 15.4	52	127.8	± 15.9	54	123.5	± 16.7	50	125.2	± 14.9	<.0001^****^	<.0001^****^	<.0001^****^	<.0001^****^	<.0001^****^
**DBP (mmHg)**	62	87.7	± 15.0	61	78.4	± 11.1	58	81.1	± 12.0	52	78.4	± 13.4	54	74.6	± 11.4	50	76.3	± 11.4	<.0001^****^	0.001^**^	0.000^***^	<.0001^****^	<.0001^****^
**RBC**	63	4.89	± 0.50	59	4.67	± 0.57	62	4.68	± 0.47	59	4.68	± 0.48	58	4.54	± 0.51	59	4.49	± 0.53	0.003^**^	<.0001^****^	<.0001^****^	<.0001^****^	<.0001^****^
**Hemoglobin**	63	14.2	± 1.58	59	13.5	± 1.79	62	13.6	± 1.45	59	13.6	± 1.63	58	13.2	± 1.78	59	13.2	± 1.99	0.002^**^	<.0001^****^	0.000^***^	<.0001^****^	<.0001^****^
**Hematocrit**	63	42.9	± 4.21	59	40.7	± 5.01	62	41.0	± 4.30	59	41.2	± 4.21	58	40.1	± 4.90	59	38.4	± 8.35	0.000^***^	<.0001^****^	0.000^***^	<.0001^****^	<.0001^****^
**WBC**	63	8.27	± 2.30	59	8.10	± 1.99	62	6.53	± 1.70	59	7.21	± 4.18	58	6.35	± 1.46	59	5.94	± 1.57	0.700	<.0001^****^	0.092	<.0001^****^	<.0001^****^
**Platelets**	63	297.2	± 62.0	59	346.5	± 74.8	62	265.8	± 65.0	58	272.9	± 58.9	58	282.9	± 74.8	59	317.2	± 329.4	<.0001^****^	<.0001^****^	0.002^**^	0.057	0.636
**AST**	63	34.9	± 20.8	58	39.7	± 22.4	62	32.9	± 17.3	59	23.4	± 11.9	58	20.2	± 6.9	59	20.0	± 8.3	0.014^*^	0.459	<.0001^****^	<.0001^****^	<.0001^****^
**ALT**	63	52.3	± 36.5	58	57.8	± 40.2	62	43.1	± 27.4	59	25.1	± 19.3	58	18.9	± 10.3	59	18.8	± 15.6	0.097	0.010*	<.0001^****^	<.0001^****^	<.0001^****^
**Total protein**	61	7.43	± 0.37	56	7.38	± 0.75	61	7.25	± 0.45	58	7.30	± 0.42	56	7.17	± 0.39	59	7.19	± 0.44	0.705	0.001^**^	0.016*	<.0001^****^	<.0001^****^
**Albumin**	63	4.38	± 0.36	58	4.25	± 0.55	62	4.28	± 0.33	59	4.29	± 0.32	57	4.28	± 0.39	59	4.31	± 0.36	0.026	0.002^**^	0.010**	0.007**	0.051
**Fasting glucose (mg/dl)**	62	123.9	± 45.3	54	102.0	± 27.1	61	105.3	± 29.1	58	101.1	± 16.9	58	98.2	± 17.8	59	95.4	± 16.6	0.000^***^	0.003^**^	0.000^***^	<.0001^****^	<.0001^****^
**Total bilirubin**	62	0.61	± 0.24	58	0.74	± 0.31	62	0.75	± 0.31	59	0.72	± 0.29	58	0.82	± 0.83	58	0.77	± 0.34	<.0001^****^	<.0001^****^	0.001^**^	0.057	<.0001^****^
**Total cholesterol (mg/dl)**	63	192.8	± 37.8	*NA*			*NA*			59	182.5	± 34.2	58	177.2	± 32.5	59	179.0	± 32.5	*NA*	*NA*	0.011	<.0001^****^	0.001^**^
**Triglyceride (mg/dl)**	63	167.5	± 135.3	*NA*			*NA*			59	115.8	± 45.2	58	101.7	± 41.5	59	90.5	± 41.3	*NA*	*NA*	0.002^**^	0.000^***^	<.0001^****^
**HDL-cholesterol (mg/dl)**	63	48.8	± 12.8	*NA*			*NA*			59	48.3	± 14.2	58	53.4	± 15.2	59	61.6	± 15.0	*NA*	*NA*	0.766	0.000^***^	<.0001^****^
**LDL-cholesterol (mg/dl)**	62	118.8	± 28.8	*NA*			*NA*			54	113.7	± 28.1	52	108.8	± 26.8	57	106.3	± 27.7	*NA*	*NA*	0.139	0.001^**^	0.000^***^
**Creatine kinase**	61	102.0	± 65.6	53	84.6	± 62.1	60	90.7	± 52.7	58	71.8	± 38.3	58	82.2	± 61.0	57	80.0	± 46.6	0.026	0.262	0.000^***^	0.114	0.004^**^
**Creatinine**	63	0.76	± 0.19	58	0.78	± 0.21	62	0.78	± 0.2	59	0.77	± 0.22	58	0.72	± 0.15	59	0.72	± 0.18	0.169	0.302	0.939	0.043	0.013^*^
**BUN**	63	12.8	± 4.70	58	12.4	± 5.66	62	10.4	± 4.7	59	11.3	± 5.15	58	12.0	± 4.46	59	13.4	± 4.17	0.634	<.0001^****^	0.016	0.093	0.351
**Uric acid**	62	6.04	± 1.87	52	8.49	± 3.50	60	6.32	± 2.2	59	5.92	± 1.82	58	5.22	± 1.53	59	5.07	± 1.46	<.0001^****^	0.192	0.654	<.0001^****^	<.0001^****^
**γ - GTP**	62	41.2	± 23.7	52	62.5	± 88.4	61	25.5	± 13.4	58	18.6	± 10.3	58	19.5	± 18.7	59	19.1	± 16.4	0.105	<.0001^****^	<.0001^****^	<.0001^****^	<.0001^****^
**Ferritin**	62	158.9	± 140.6	*NA*			*NA*			59	143.3	± 133.3	57	111.7	± 107.6	55	89.9	± 108.1	*NA*	*NA*	0.137	0.001	<.0001^****^
**Vitamin B12**	63	533.6	± 217.3	*NA*			*NA*			58	539.6	± 290.9	56	459.4	± 194.1	56	462.8	± 244.8	*NA*	*NA*	0.820	0.001^**^	0.042^*^

Data are presented as the mean ± SD. N/A denotes not available. The values at each postoperative time point were compared with preoperative values. 1) Paired samples t-test (preoperative to 1 week), 2) paired samples t-test (preoperative to 1 month), 3) paired samples t-test (preoperative to 3 months), 4) paired samples t-test (preoperative to 6 months), and 5) paired samples t-test (preoperative to 12 months); *p < 0.5, **p < 0.1, ***p < 0.001, ****p < 0.0001.

Twelve months after surgery, liver function was also improved in terms of aspartate aminotransferase (AST), alanine transaminase (ALT), and gamma-glutamyl transferase (γGTP) levels. Lipoprotein profiles were also improved after BS, showing lower triglyceride (p<0.001), total cholesterol (p<0.001), and low-density lipoprotein (LDL) cholesterol (p=0.000) and increased high-density lipoprotein (HDL) cholesterol levels (p<0.001) at the 12-month follow-up compared to baseline. These significant weight losses also led to a continued decrease in fasting glucose levels.

**Figure 1:**
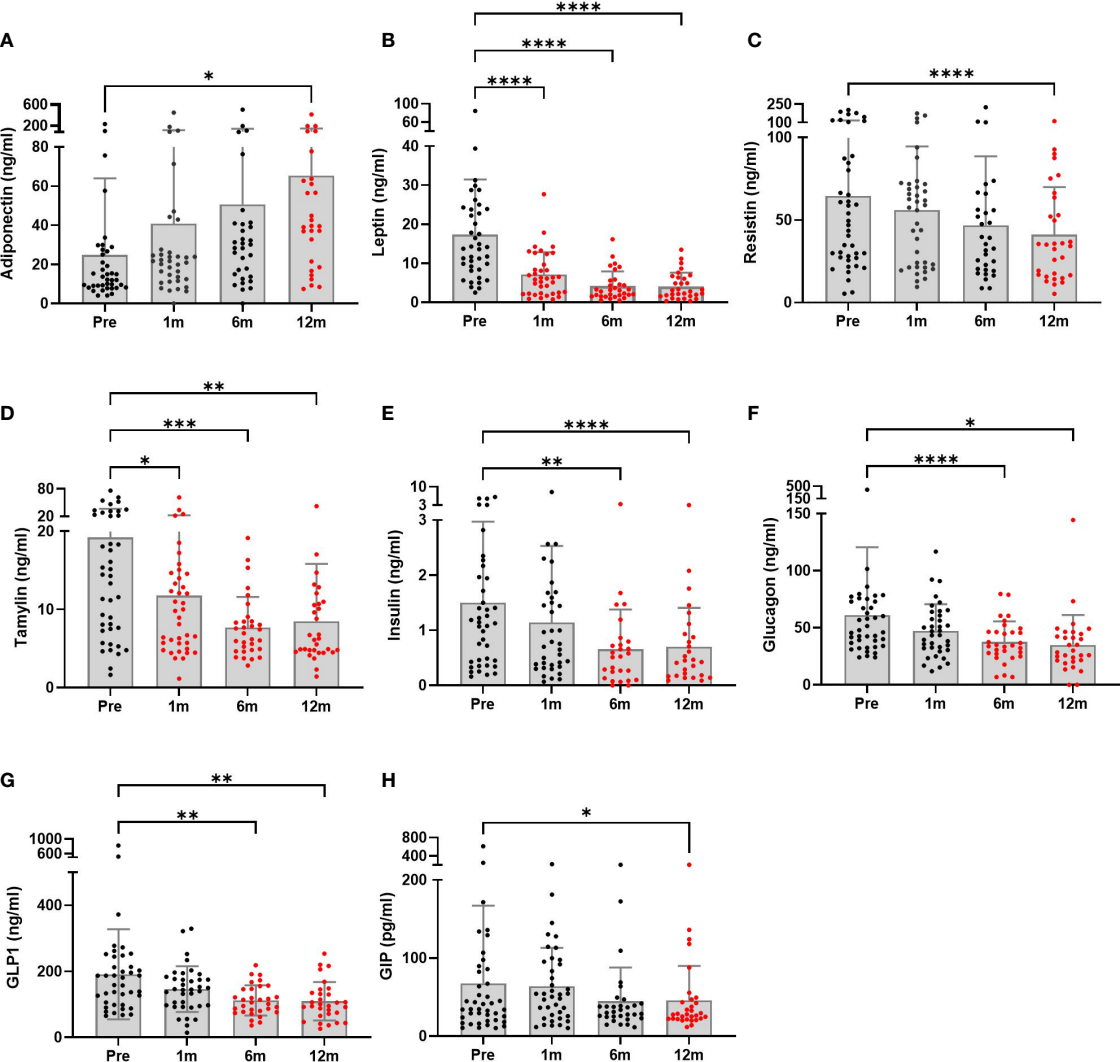
Metabolic hormone profiles of patients undergoing bariatric surgery (n = 28 to 40) were monitored at baseline and postoperative time points. **(A–C)** Adipose tissue hormones, **(D–F)** pancreatic hormones, and **(G, H)** gastrointestinal hormones. The graphs are presented showing means and SDs. Significant differences between baseline and each postoperative time point were determined; *p < 0.5, **p < 0.1, ***p < 0.001, ****p < 0.0001. Pre, pre-surgery; 1m, 1month post-surgery; 3m, 3 months post-surgery; 12m, 12 months post-surgery.

Hormones secreted by adipose tissue (adiponectin, leptin, and resistin) showed significant changes postoperatively (**Figure 1**). Adiponectin levels were increased significantly at the 12-month follow-up compared to baseline (40.51 ± 15.61 ng/ml mean increment, p=0.041). Leptin levels were significantly decreased from 1 month after surgery. A decreasing pattern of resistin was seen, and the difference compared to baseline finally became significant at 12 months (23.55 ± 4.66 ng/ml mean reduction, p<0.0001). Pancreatic hormone levels showed continuous reductions over 12 months. Compared to baseline levels, surgery was associated with significantly decreased amylin levels as early as 1 month post-surgery (7.44 ± 2.79 ng/ml mean reduction, p=0.031) which was maintained up to 12 months (10.72 ± 2.69 ng/ml, p=0.001). Insulin and glucagon levels were also greatly reduced at 6 months postoperatively. As previously reported (23), BS markedly lowered the levels of gut-derived incretin hormones GLP-1 at 6 and 12 months and GIP at 12 months.

**Figure 2:**
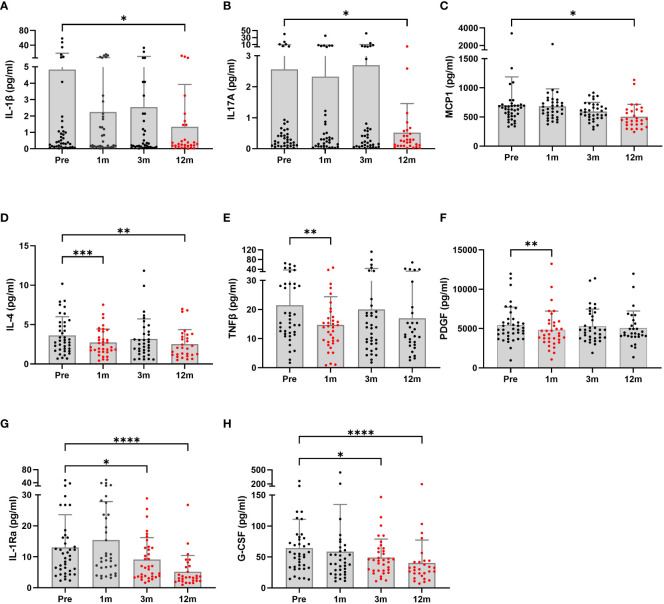
Serum inflammatory profiles of patients undergoing bariatric surgery (n = 29 to 40) were monitored at baseline and postoperative time points. The graphs are presented as the means and SDs. Significant differences between baseline and postoperative time points were determined; *p < 0.5, **p < 0.1, ***p < 0.001, ****p < 0.0001. Pre, pre-surgery; 1m, 1month post-surgery; 3m, 3 months post-surgery; 12m, 12 months post-surgery.

**Figure 2** shows the changes in serum inflammatory cytokines at follow-up. IL-1β, IL-17A, and MCP-1 levels were significantly decreased at the 12-month follow-up compared to baseline (p=0.037, p=0.011 and p=0.050, respectively). An inconsistency was noted in cytokine levels. IL-4 levels were significantly decreased at the first 1-month (p=0.0001) and last 12-month follow-up (p=0.003). However, improvements in TNF-β and PDGF levels that occurred during the first month after surgery were not sustained. Lastly, IL-1Rα and G-CSF levels were much lower, starting 3 months postoperatively.

### 3.3 Diabetic patients show differences in type 2 diabetes remission 12 months after bariatric surgery

Of the 63 patients who underwent bariatric surgery, 23 patients were clinically diagnosed with diabetes at baseline. The demographics and clinical characteristics of the remission and the non-remission groups at baseline are shown in **Table 3**. There were significant differences in age and waist-hip ratio (WHR) between the two groups, based on only the t-test (p = 0.014 and 0.011, respectively).

**Table 3:** Baseline characteristics of patients with and without type 2 diabetes remission 12-months after bariatric surgery.

	Remission (*N*=11)			No diabetes remission (*N*=12)			*p*-value^1)^	*p*-value^2)^
Sex, n(%)	11			12			1.000	1.000
Men		3	(27.3)		3	(25.0)		
Women		8	(72.7)		9	(75.0)		
Age (years)	11	38.1	± 11.3	12	50.0	± 9.9	0.014*	0.029
Weight (kg)	11	105.2	± 29.5	12	98.6	± 23.5	0.560	0.693
BMI (kg/m^2^)	11	36.7	± 5.0	12	38.7	± 8.7	0.518	0.903
Waist circumference (cm)	10	113.6	± 16.2	12	118.3	± 14.6	0.481	0.349
WHR(Waist-Hip Ratio)	10	0.96	± 0.05	12	1.02	± 0.04	0.011*	0.017
SBP (mmHg)	11	141.5	± 21.1	12	139.2	± 12.1	0.750	0.784
DBP (mmHg)	11	84.5	± 14.5	12	87.8	± 8.7	0.523	0.230
Fasting glucose (mg/dl)	11	165.1	± 59.2	12	158.3	± 54.5	0.776	0.784
Total cholesterol (mg/dl)	11	186.8	± 33.6	12	197.8	± 50.8	0.550	0.585
Triglyceride (mg/dl)	11	194.5	± 119.1	12	247.3	± 256.6	0.530	0.855
HDL-cholesterol (mg/dl)	11	48.4	± 11.5	12	47.4	± 10.4	0.838	0.903
LDL-cholesterol (mg/dl)	11	117.5	± 28.0	12	114.1	± 31.9	0.791	0.855
Hypertension, n(%)	11	7	(63.6)	12	11	(91.7)	0.155	0.155
Dyslipidemia, n(%)	11	9	(81.8)	12	9	(75.0)	1.000	1.000
Bariatric surgery, n(%)	11			12			1.000	1.000
Roux-e-Y Gastric Bypass		5	(45.5)		5	(41.7)		
Sleeve Gastrectomy		6	(54.6)		7	(58.3)		

Twenty-three patients diagnosed were T2D before surgery were split into two groups according to diabetes remission at the 12-month follow-up. Values are presented as the mean ± SD for continuous variables and n (%) for categorical variables. 1) Fisher’s exact test or t-test, 2) Fisher’s exact test or Wilcoxon’s rank sum test.

Demographics and clinical characteristics were compared between baseline and the 12-month follow-up point in each remission and non-remission group to investigate the effect of BS on T2D improvement (**Tables 4**, **5**). In both remission and non-remission groups, the parameters with significant and non-significant changes are shown in **Table 4**. Notably, vitamin B12 showed a significant reduction 12 months postoperatively only in the non-remission group. Twelve-month postoperative white blood cell and platelet counts, and fasting glucose, AST, ALT, TG, LDL, uric acid, γGTP, and thyroid-stimulating hormone (TSH) levels were also significantly reduced from the preoperative values only in the remission group (**Table 5**). Total bilirubin levels were slightly increased in both groups, but statistical significance before and after surgery was found only in the remission group.

**Table 4:** Comparison of clinical characteristic before and 12 months after bariatric surgery.

	Remission (n=11)										Non-remission (n=12)									
	Baseline			Postoperative 12 months			Change over 12-month			p-value	Baseline			Postoperative 12 months			Change over 12-month			p-value
	N	Mean	±SD	N	Mean	±SD	N	Mean	±SD		N	Mean	±SD	N	Mean	±SD	N	Mean	±SD	
**Weight** (kg)	11	105.2	± 29.5	11	71.4	± 18.1	11	-33.8	± 14.7	<.0001^****^	12	98.6	± 23.5	9	72.9	± 17.8	9	-19.1	± 11.1	<0.001^***^
**BMI** (kg/m^2^)	11	36.7	± 5.0	11	25.1	± 3.5	11	-11.7	± 3.5	<.0001^****^	12	38.7	± 8.7	9	28.2	± 4.9	9	-7.6	± 4.8	0.001^**^
**Waist circumference** (cm)	10	113.6	± 16.2	10	87.7	± 13.4	10	-25.9	± 9.0	<.0001^****^	12	118.3	± 14.6	9	94.4	± 12.2	9	-19.8	± 9.8	<0.001^***^
**Hip**	10	117.9	± 14.2	10	97.5	± 10.0	10	-20.4	± 7.6	<.0001^****^	12	116.2	± 14.5	9	101.8	± 11.4	9	-9.7	± 9.7	0.017^*^
**WHR**(Waist-Hip Ratio)	10	0.96	± 0.05	10	0.90	± 0.07	10	-0.07	± 0.07	0.021^*^	12	1.02	± 0.04	9	0.93	± 0.06	9	-0.10	± 0.05	<0.001^***^
**SBP** (mmHg)	11	141.5	± 21.1	10	117.4	± 11.5	10	-25.2	± 15.6	<0.001^***^	12	139.2	± 12.1	9	124.3	± 12.1	9	-11.2	± 12.5	0.028^*^
**DBP** (mmHg)	11	84.5	± 14.5	10	72.4	± 7.1	10	-13.6	± 11.3	0.004^**^	12	87.8	± 8.7	9	75.3	± 9.7	9	-10.8	± 11.8	0.025^*^
**RBC**	11	5.00	± 0.40	11	4.52	± 0.36	11	-0.48	± 0.42	0.004^**^	12	4.91	± 0.66	10	4.40	± 0.69	10	-0.53	± 0.43	0.004^**^
**Hemoglobin**	11	14.6	± 1.3	11	13.3	± 1.7	11	-1.3	± 1.7	0.032^*^	12	13.8	± 1.8	10	12.5	± 2.4	10	-1.5	± 1.5	0.012^*^
**Chloride(CI)**	11	100.8	± 2.3	10	102.9	± 3.2	10	1.9	± 2.3	0.027^*^	12	101.4	± 3.7	10	103.5	± 2.6	10	2.0	± 2.7	0.047^*^
**MCV**	11	87.3	± 2.8	11	87.0	± 5.6	11	-0.3	± 4.6	0.842	12	86.5	± 5.9	10	86.5	± 8.0	10	-0.3	± 4.6	0.826
**MCH**	11	29.2	± 1.3	11	29.3	± 2.7	11	0.1	± 1.9	0.832	12	28.3	± 2.4	10	28.4	± 3.3	10	-0.1	± 2.0	0.857
**MCHC**	11	33.4	± 1.1	11	33.6	± 1.5	11	0.2	± 1.0	0.537	12	32.7	± 0.9	10	32.7	± 1.5	10	-0.1	± 1.1	0.864
**RDW**	10	12.9	± 0.8	9	14.2	± 3.6	9	1.4	± 3.4	0.247	10	13.6	± 1.3	5	13.8	± 1.5	5	0.6	± 0.5	0.052
**Total protein**	11	7.45	± 0.38	11	7.38	± 0.52	11	-0.07	± 0.66	0.721	12	7.44	± 0.38	10	7.25	± 0.42	10	-0.22	± 0.34	0.071
**Albumin**	11	4.54	± 0.38	11	4.49	± 0.42	11	-0.05	± 0.49	0.767	12	4.25	± 0.31	10	4.30	± 0.31	10	-0.03	± 0.22	0.678
**Alk. Phosphatase**	11	104.9	± 78.6	11	86.2	± 43.1	11	-18.7	± 41.8	0.168	12	99.4	± 41.1	10	95.6	± 35.3	10	-4.1	± 27.8	0.652
**Potassium(K)**	11	4.07	± 0.34	10	4.08	± 0.56	10	-0.06	± 0.67	0.783	12	4.50	± 0.59	10	4.26	± 0.44	10	-0.26	± 0.49	0.126
**Total cholesterol** (mg/dl)	11	186.8	± 33.6	11	170.5	± 37.7	11	-16.3	± 25.2	0.058	12	197.8	± 50.8	10	199.6	± 36.4	10	-3.7	± 41.1	0.782
**HDL-cholesterol** (mg/dl)	11	48.4	± 11.5	11	60.2	± 13.2	11	11.8	± 12.5	0.010*	12	47.4	± 10.4	10	57.8	± 11.1	10	13.0	± 12.8	0.010*
**Calcium**	11	9.44	± 0.27	11	9.32	± 0.57	11	-0.12	± 0.62	0.539	12	9.43	± 0.39	10	9.31	± 0.44	10	-0.14	± 0.36	0.246
**Creatine kinase**	11	83.6	± 45.8	10	65.6	± 34.0	10	-15.2	± 21.9	0.056	12	110.8	± 58.3	10	84.2	± 58.2	10	-25.6	± 49.9	0.139
**Creatinine**	11	0.67	± 0.14	11	0.67	± 0.13	11	-0.002	± 0.13	0.964	12	0.78	± 0.24	10	0.77	± 0.30	10	-0.02	± 0.13	0.628
**BUN**	11	11.4	± 3.2	11	13.6	± 3.9	11	2.2	± 3.4	0.058	12	14.7	± 7.9	10	15.9	± 6.8	10	1.0	± 3.1	0.318
**Iron**	11	119.3	± 82.1	11	113.0	± 83.2	11	-6.3	± 46.5	0.664	11	88.4	± 28.4	8	100.5	± 56.9	7	5.0	± 48.8	0.795
**Vitamin B12**	11	516.0	± 167.4	11	538.0	± 352.1	11	22.0	± 418.8	0.865	12	678.4	± 339.8	9	524.7	± 356.8	9	-157.5	± 189.2	0.037*
**Free T4**	11	1.22	± 0.19	10	1.21	± 0.22	10	-0.01	± 0.14	0.878	12	1.23	± 0.26	9	1.16	± 0.18	9	-0.09	± 0.09	0.016*
**T3**	11	49.3	± 66.9	10	42.3	± 56.0	10	-11.8	± 22.3	0.127	12	19.7	± 45.4	9	10.3	± 28.3	9	-6.6	± 19.3	0.332
**PTH**	11	35.5	± 15.0	10	48.2	± 19.6	10	14.4	± 21.1	0.059	12	51.0	± 14.7	8	48.8	± 6.9	8	-2.6	± 8.5	0.406

Data are presented as the mean ± SD. Significant differences between baseline and 12 months after surgery in remission and non-remission groups were determined using the paired t-test; *p < 0.5, **p < 0.1, ****p < 0.0001.

**Table 5:** Impact of bariatric surgery on diabetes remission at the 12th month after bariatric surgery.

	Remission (n=11)										Non-remission (n=12)									
	Baseline			Postoperative 12 months			Change over 12-month			p-value	Baseline			Postoperative 12 months			Change over 12-month			p-value
	*N*	Mean	±SD	*N*	Mean	±SD	*N*	Mean	±SD		*N*	Mean	±SD	*N*	Mean	±SD	*N*	Mean	±SD	
**Fasting glucose** (mg/dl)	11	165.1	± 59.2	11	96.9	± 12.3	11	-68.2	± 55.1	0.002^**^	12	158.3	± 54.5	10	114.3	± 29.5	10	-47.6	± 70.2	0.061
**WBC**	11	9.33	± 2.73	11	6.33	± 2.12	11	-3.00	± 2.62	0.004^**^	12	8.01	± 2.83	10	6.39	± 2.24	10	-1.21	± 2.30	0.130
**Platelets**	11	291.9	± 70.2	11	257.2	± 73.0	11	-34.7	± 35.4	0.009^**^	12	292.8	± 83.2	10	283.5	± 143.5	10	-2.7	± 62.7	0.895
**AST**	11	34.9	± 17.9	11	21.9	± 7.7	11	-13.0	± 13.4	0.009^**^	12	35.4	± 19.4	10	26.1	± 14.8	10	-11.8	± 20.0	0.095
**ALT**	11	58.0	± 30.7	11	24.3	± 19.4	11	-33.7	± 39.2	0.017^*^	12	41.4	± 24.6	10	26.7	± 27.5	10	-18.1	± 38.6	0.173
**Total bilirubin**	11	0.62	± 0.24	11	0.81	± 0.40	11	0.19	± 0.26	0.032^*^	12	0.65	± 0.21	10	0.82	± 0.38	10	0.15	± 0.31	0.175
**Triglyceride** (mg/dl)	11	194.5	± 119.1	11	92.8	± 42.0	11	-101.6	± 111.1	0.013^*^	12	247.3	± 256.6	10	121.9	± 65.0	10	-154.6	± 262.6	0.096
**LDL-cholesterol** (mg/dl)	11	117.5	± 28.0	11	99.6	± 26.9	11	-17.8	± 17.1	0.006^**^	12	114.1	± 31.9	10	130.8	± 31.1	10	11.4	± 29.3	0.250
**Uric acid**	11	5.83	± 1.65	11	4.94	± 1.24	11	-0.89	± 0.96	0.012^*^	12	5.33	± 1.50	10	4.87	± 1.48	10	-0.30	± 1.28	0.478
**γ - GTP**	11	39.4	± 16.4	11	15.7	± 6.1	11	-23.7	± 12.6	<.0001^****^	12	47.8	± 30.6	10	34.3	± 32.0	10	-13.0	± 40.1	0.332
**TSH**	11	2.12	± 1.14	10	1.44	± 1.16	10	-0.66	± 0.76	0.022^*^	12	2.75	± 1.93	9	2.20	± 0.93	9	-0.76	± 1.70	0.217

Data are presented as the mean ± SD. Significant differences between baseline and 12 months after surgery in the remission and non-remission groups were determined using the paired t-test; *p < 0.5, **p < 0.1, ****p < 0.0001.

**Figure 3:**
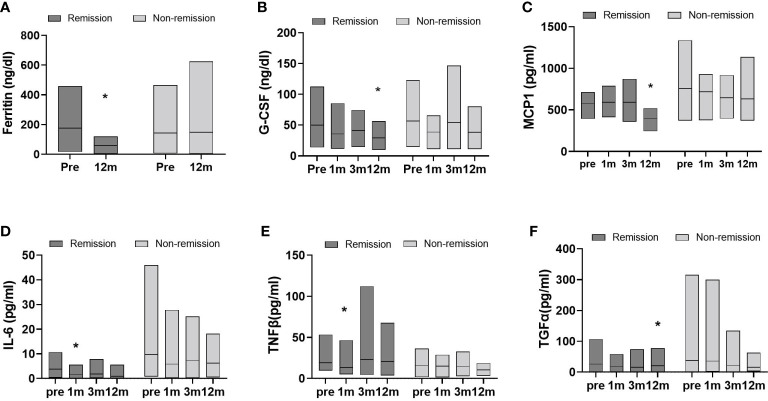
Data represent as a floating bar (min to max value) with a line indicating the mean of cytokine levels. Significant differences between baseline and postoperative time points within group were determined (remission group, n=11; non-remission group, n=12); *p < 0.5, Pre, pre-surgery; 1m, 1month post-surgery; 3m, 3 months post-surgery; 12m, 12 months post-surgery.

**Figure 4:**
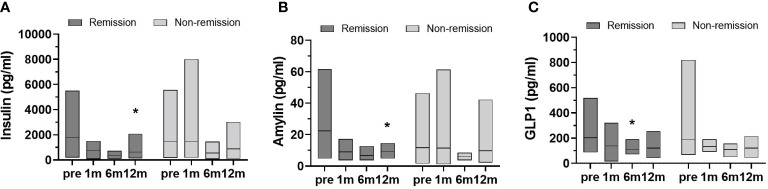
Data represent as a floating bar (min to max value) with a line indicating the mean of hormone levels. Significant differences between baseline and postoperative time points within group were determined (remission group, n=11; non-remission group, n=12); *p < 0.5. Pre, pre-surgery; 1m, 1month post-surgery; 6m, 6 months post-surgery; 12m, 12 months post-surgery.

Changes in inflammatory cytokine levels were analyzed in both groups at follow-ups after surgery (**Figure 3**). Statistical significances for postoperative time points were only seen in the remission group. At 12 months, for example, G-CSF, MCP-1, and TGFα levels were markedly decreased compared to baseline. Significant reductions in IL-6 and TNFβ levels occurred the first month after surgery and were not sustained. Hormone changes were also assessed over time after surgery. The remission group showed significant changes in insulin, amylin, and GLP-1 levels (**Figure 4**). Insulin and amylin showed continuously decreasing patterns after surgery, and statistical significance was found at 12 months. Although GLP-1 continued to decrease over time in both groups, a significant reduction was only found in the remission group at 6 months after surgery.

## 4 Discussion

In this study we found that, among Korean patients with obesity, adiponectin increased and leptin, resistin, total amylin, insulin, glucagon, GLP1, and GIP decreased over 12 months following BS. These hormone changes accompany with ameliorating inflammatory cytokine levels in serum, such as IL-1β, IL-17A, and MCP1. We further divided the patients into postoperative diabetes remission and non-remission subgroups and found that remission group significantly reduced the serum levels of insulin, amylin and GLP1 hormone and several inflammatory cytokines including MCP1 and IL-6.

First, we observed a significant increase in adiponectin levels after BS in patients with obesity. It has been reported that, in patients with morbid obesity, abnormal levels and functioning of adipokines were detected. The levels of other adipokines, leptin and resistin, decreased. In obesity, increased visceral fat results in lower adiponectin and increased leptin levels (24). A large reduction in body fat through BS improves metabolic abnormalities in patients with obesity (25). High adiponectin levels were accompanied by a significant reduction in leptin levels (26). Leptin resistance is a well-known characteristic of metabolic disorders (27). Moreover, enhancing adiponectin levels improved metabolic syndrome independent of body weight (28). In our study, leptin levels dropped at 1 month postoperatively and were sustained until 12 months postoperatively, whereas adiponectin levels gradually increased and reached significance at 12 months. These changes were observed with lower insulin and glucagon levels, indicating improvement in glucose metabolism.

In a cross-sectional study using The Danish Childhood Obesity Data and Biobank data, plasma GLP1 concentrations were associated with the degree of obesity and metabolic disorders (6). GLP1 is expressed in L-cells in the distal jejunum and ileum, and gastric inhibitory polypeptide (GIP) is produced from enteroendocrine cells in the proximal gut. These hormones control glucose homeostasis and gut motility (29, 30). GLP-1 receptor signaling enhances β-cell glucose metabolism through mammalian target of rapamycin (31).

In our study, postoperative incretin hormone levels (amylin, GIP, and GLP1) were reduced with lower glucose, glucagon, and insulin levels. Postprandial GLP1 levels after RYGB and SG cannot be achieved with weight loss due to caloric restriction (32). These data suggest that BS benefits on improvement of metabolic profiles are not only due to reduction of body weight, but also changes in food intake and metabolic hormone levels.

In terms of inflammation, the production of IL-4 by lymphocytes increased serum levels in obese mice (33). Furthermore, TNFβ controls responses to diet-induced obesity (34), and it is responsible for early inflammatory reactions, cytotoxicity, and apoptosis (35–37). Severe obesity expands adipose tissue, through which PDGF-B-dependent vascular remodeling occurs (36). IL-4, TNFβ, and PDGF levels dropped in the early stages after BS, and other proinflammatory cytokines associated with obesity, such as Il-1β, IL-17A, MCP1, IL-1Ra, and G-CSF, gradually decreased over 1 year postoperatively. In addition to that, liver damage markers, ALT, AST, and γGTP were significantly ameliorated a month postoperatively. This suggests that the inflammatory status is ameliorated through sequential changes in inflammatory factors originated from different organ sites.

Next, we divided the patients into diabetes remission and non-remission groups. The effect of BS on body weight, BMI, and hypertension significantly improved in both groups, and total cholesterol and HDL did not significantly change in both groups. It seems the difference remission or non-remission comes from how improved inflammatory factors since there was a significant difference between inflammatory factor levels. Elevated serum ferritin levels are an important indicator and predictor of the incidence of T2D and insulin resistance (38–40) and are associated with clinical inflammation (41, 42). In our study, the remission group showed significant decreases in factors associated with monocyte migration, inflammatory proteins, and insulin resistance-related cytokines, as illustrated by the changes in G-CSF, MCP1, and TNFβ (43–46). These results, accompanied by reduced insulin or incretin hormone levels, were only significant in the remission group. The data showing the lack of significant changes in the non-remission T2D group suggests that hormones, immunological factors, and inflammatory vigor play a dominant role in metabolic dysfunction in the obese subpopulation rather than body weight itself. Notably, the non-remission group had high levels of IL-6 and TGFα and had more variations in values within the group compared to the remission group, indicating the degree of reduction in these two cytokines after BS may be crucial in the remission of diabetes.

This study had several strengths and limitations. First, because few studies to date have examined the effect of BS in Korean patients, this study significantly contributes to the field. Second, this novel cohort reproduced the results of previous studies that were conducted in other groups with different ethnic and geographical backgrounds. Third, the BS group was classified into subpopulations with type 2 diabetes. However, owing to the small number of patients, they could not be divided into subgroups according to the type of surgery (Roux-en-Y and gastric sleeve) for a more detailed analysis. The effect of BS differs according to age and duration of diagnosis. Results in this study need to be confirmed in a study with a larger population.

The effects of BS in Korean patients are not well-known. Our study analyzed changes in blood parameters postoperatively. The changes in hormones secreted from the three major metabolic tissues (pancreas, adipose, and gut) and differences in multi-origin inflammatory cytokines between remission and non-remission groups provide a path for understanding how the effect of BS in improving glucose metabolism is mediated. The accumulated data will provide valuable information for the development of noninvasive anti-obesity and metabolic syndrome marker that are more appropriate for Korean patients.

## Data availability statement

The original contributions presented in the study are included in the article/Supplementary Material. Further inquiries can be directed to the corresponding author.

## Ethics statement

The studies involving human participants were reviewed and approved by Korea Disease Control and Prevention Agency (KDCA) Inha University Hospital Institutional Review Board. The patients/participants provided their written informed consent to participate in this study.

## Author contributions

EK, JY, and SK designed the research. JY, H-JK, YH, DP, and YP contributed to acquisition of data. EK, JY, H-JK, and HP analyzed data and EK wrote the manuscript. All authors listed have made a substantial, direct, and intellectual contribution to the work and approved it for publication.
